# Effects of Kaixinjieyu, a Chinese herbal medicine preparation, on neurovascular unit dysfunction in rats with vascular depression

**DOI:** 10.1186/s12906-015-0808-z

**Published:** 2015-08-19

**Authors:** Juhua Pan, Xiaoming Lei, Jialong Wang, Shijing Huang, Yanyun Wang, Ying Zhang, Wen Chen, Duojiao Li, Jun Zheng, Hanming Cui, Qihua Liu

**Affiliations:** Guang’anmen Hospital, China Academy of Chinese Medical Sciences, Beijing, 100053 China; Huangpi District Hospital of Traditional Chinese Medicine, Wuhan, Hubei 430300 China

**Keywords:** Kaixinjieyu, Chinese herbal medicine, Vascular depression, Neurovascular unit, Blood–brain barrier, Neurogenesis

## Abstract

**Background:**

Kaixinjieyu (KJ), derived from Kaixin and Sini powder, is an effective Chinese herbal medicine preparation used in the treatment of vascular depression (VD). We hypothesize that broad antidepressant effect of KJ results from the improved neurovascular unit (NVU) function via neurogenesis, permeability of blood–brain barrier (BBB) and balance of the fibrinolytic system.

**Methods:**

A VD model of rat was established by chronic unpredictable mild stress and separation after ligation of the bilateral common carotid arteries. The rats were treated with KJ and fluoxetine hydrochloride (FLU) for 21 days, respectively. The behavior and cerebral perfusion were investigated and then NVU functions including neurogenesis, permeability of BBB and balance of the fibrinolytic system were studied using a number of biomarkers and TUNEL assay.

**Results:**

KJ significantly increased sucrose preference, moving distance, number of rearing and cortical blood flow. NVU functions measured by brain-derived neurotrophic factor (BDNF), tropomyosin receptor kinase B (TrkB) and tissue plasminogen activator (t-PA) proteins and mRNA, zona occludens protein-1 (ZO-1), occludin and claudin-5 proteins increased significantly, whereas, plasminogen activator inhibitor-1 (PAI-1), matrix metalloproteinase-2 (MMP-2) proteins, mRNA and apoptotic rates of neurons decreased significantly with treatment of KJ. FLU has a function similar to KJ in behavior, regulation of BDNF, TrkB, MMP-2, occludin and apoptotic rates of cells.

**Conclusions:**

KJ has function of reducing depression-like behavior and improving cerebral hypoperfusion, which might be mediated by the up-regulation of neurogenesis and tight junction of BBB, and balance of the fibrinolytic system. The results imply that KJ is better than FLU in improving cerebral hypoperfusion and the fibrinolytic system.

## Background

Vascular depression (VD) is defined as a subtype of late-life depression associated with vascular diseases or cerebrovascular risk factors such as age, stroke, myocardial infarction, hypertension, hyperlipidemia and diabetes [1, 2]. Studies have shown that about 3.4 % adults of 50-years and older suffer from VD. Among adults who meet the criteria for lifetime major depression, over one-in-five are considered to have the VD subtype [3]. VD has three subtypes, namely, post-stroke depression, MRI-defined VD and depression with cerebrovascular disorder risk factors [4]. Their common pathology is chronic cerebral ischemia caused by cerebrovascular diseases [5]. The event of stressful life notably results in the occurrence of VD [6].

A number of associated diseases of VD means complicated therapeutic strategies [1, 7]. For VD patients, depressive syndrome is not the primary disease but can be considered as one of the clinical manifestations in the wide symptom spectrum of the cerebral small vessel diseases [7]. VD is poorly responsive to antidepressants and the therapeutic strategy is often significantly different from other depressions. Therefore, VD is a useful concept for clinical interventions [8]. White-matter hyper intensities in the left hemisphere [9], especially in the cingulum bundle adjacent to the hippocampus [10], global vascular risk [11], magnetic resonance imaging findings and neuropsychological assessment [12] have been used as predictors of poor response to antidepressants in VD patients.

Earlier studies have shown that monoamine and its receptors are likely to have antidepressant-like effects only at the beginning, and subsequently, it is neural plasticity that generates the long-lasting antidepressant effects [13]. The current hypothesis of depression points toward neuroplasticity-related malfunction in information processing within neural networks regulating mood [14, 15]. Neural plasticity and neurogenesis have become one of the main targets of antidepressants. Gudmundsson et al. reported that elderly women with major depressive disorder exhibited higher cerebrospinal fluid /serum albumin ratios compared to women without depression which indicates that blood–brain barrier (BBB) hyper-permeability occurs in major depressive disorder [16]. Increasingly the BBB, which is formed by endothelial cells, is no longer viewed as an isolated conception, but as a major component of an integrated neurovascular unit (NVU) [17]. The NVU consists of neurons, endothelial cells, pericytes and glia, which are tightly coupled to control cerebrovascular function. This structural and functional unit may have major roles in VD pathogenesis [18]. Restoration of NVU function is considered as one of the main therapeutic targets.

The previous research has shown that Kaixinjieyu (KJ), a Chinese herbal medicine preparation from Kaixin and Sini powder, is capable of enhancing vital Qi (Chinese words, meaning the function of body and the ability of health maintenance) and reducing depression; it could improve both cognitive and physical function of VD patients clinically [19]. KJ has antidepressant effects on behavior and production of monoamines such as the neurotransmitters serotonin and norepinephrine and serotonin receptor of chronic unpredictable mild stress (CUMS) in rats [19]. Moreover, the previous study has demonstrated that KJ can increase the expression of glial fibrillary acidic protein and brain-derived neurotrophic factor (BDNF) in the hippocampus in VD model [20]. However, the effect of KJ on the dysfunction of neurovascular unit in VD is still unclear, especially in the prefrontal cortex and hippocampus, which are central in the pathophysiology of mood disorders [14].

In the present study, the antidepressant effects and a number of biomarkers of NVU functions including neurogenesis, fibrinolytic system and permeability of BBB were investigated in KJ-treated VD rats, such as BNDF, tropomyosin receptor kinase B (TrkB), tissue plasminogen activator (t-PA), plasminogen activator inhibitor-1 (PAI-1), matrix metalloproteinase-2 (MMP-2), matrix metalloproteinase-9 (MMP-9), zona occludens protein-1 (ZO-1), occludin and claudin-5 [21–23].

## Methods

### Preparation and compositional analysis of KJ

KJ is a prescription originated from eight herbal plants including *Bupleurum chinense* DC. (Chai-Hu), *Paeonia lactiflora* Pall. (Chi-Shao), *Morinda officinalis* How (Ba-Ji-Tian), *Poria cocos* (Schw.) Wolf (Fu-Ling), *Panax ginseng* C. A. Mey. (Ren-Shen), *Citrus aurantium* L.(Zhi-Shi), *Polygala tenuifolia* Willd. (Yuan-Zhi) and *Glycyrrhiza uralensis* Fisch. (Gan-Cao), in weight ratio of 3:3:3:3:2:2:2:2. These plant materials were bought from Beijing Bencao Fangyuan Pharmaceutical Co., Ltd (Beijing, China). *Panax ginseng* C. A. Mey. and *Poria cocos* (Schw.) Wolf were grinded into powder of less than 100 meshes in size. Other herbs were decocted twice, each for 1.5 h. The filtrates were merged and concentrated to obtain a cream (relative density of 1.30–1.35 g/cm^3^ at 55–60 °C) by evaporation under reduced pressure. The preparation (KJ) was made up with the powder and cream by weight ratio of 1.2:1, and analyzed for composition by high performance liquid chromatography (HPLC).

The test solution was prepared by dissolving KJ in methanol, and analyzed on an Agilent 1200 HPLC (DAD) system with an C18 analytical column (250 × 4.6 mm, 5 μm). The mobile phase was consisted of acetonitrile and water in gradient elution. For quality control, standards including ginsenosides Rg_1_, Re and Rb_1_ for *Panax ginseng* C. A. Mey., nistose for *Morinda officinalis* How, paeoniflorin for *Paeonia lactiflora* Pall., saikosaponin A and D for *Bupleurum chinense* DC., Glycyrrhizic acid ammonium salt and liquiritin for *Glycyrrhiza uralensis* Fisch.*,* naringin, neohesperidin and hesperidin for *Citrus aurantium* L. were used. All the standards were purchased from National Institutes for Food and Drug Control (Beijing, China).

### Animal, model and treatment

Eighty male Sprague–Dawley rats (250 ± 10 g, 7 weeks old), SPF grade, were purchased from Vital River Lab Animal Technology Co., Ltd (Beijing, China). All experiments were approved by the Ethical Committee of Guang’anmen Hospital, China Academy of Chinese Medical Sciences (Beijing, China). The animals were cared in accordance with the “Guide for the Care and Use of Laboratory Animals” of the National Institutes of Health. Rats were initially divided into two groups: sham-operated rats (sham group, *n* = 8) were fed in a breeding box each with 4 rats normally and a permanent ligation of bilateral common carotid arteries (LBCCA) group (*n* = 72). The rats were anesthetized with intraperitoneal chloral hydrate (350 mg/kg). Through a midline cervical incision, the bilateral common carotid arteries were exposed and double ligated with silk sutures. The sham-operated rats were treated in a manner similar to that of the operated ones, except that the common carotid arteries were not occluded [24]. Forty-two survived rats were randomly divided into 5 groups for sucrose preference test (SPT) and fed individually in a box and received CUMS for 21 consecutive days from 7 days post LBCCA. Some were treated with KJ at dose of 1.8 g/kg/day (KJ-H, *n* = 9), 0.9 g/kg/day (KJ-M, *n* = 9), 0.45 g/kg/day (KJ-L, *n* = 8) and fluoxetine hydrochloride at dose of 2.0 mg/kg/day (FLU, *n* = 8). KJ and FLU were administered for 21 consecutive days from 7 days post LBCCA. The others were treated with nothing as VD model (VD, *n* = 8) [20]. Mild stressors used included placing an empty bottle in cages for 1 h, nipping the tail for 1 min, swimming in 4 °C water for 5 min, heating in 45 °C oven for 5 min, inversion of the light/dark cycle for 24 h, cage tilting for 24 h, fasting for 24 h, water deprivation for 24 h, wet bedding for 24 h, shaking for 3 min (160 rpm) and electric shock (60 V, 1 mA, lasting 10 s at an interval of 1 min, 5 times). Rats received one of these stressors per day, but the same stressor was not applied in two consecutive days.

### Behavior tests

SPT and open-field test (OFT) were carried out after CUMS as described [25]. The consumptions of 1 % sucrose solution and distilled water in 2 h were measured. The sucrose preference was calculated as the percentage of the sucrose solution consumed to the solution (sucrose solution and water) consumed. OFT was tested using the OFT-100 video analysis system. A rat was placed in the center of the box individually and recorded for its moving distance and the number of rearing.

### Cerebral perfusion

All rats were anesthetized with 10 % chloral hydrate (350 mg/kg) after the behavioral tests. Skin was removed and skull fully exposed. The cerebral perfusion was captured with a Perfusion Speckle Imager (Perimed AB, Sweden). Data were processed using PIMSoft and the means of cortical blood flow (CBF) in the left and right hemispheres were calculated.

### The terminal transferase dUTP nick end labeling (TUNEL) assay

Rats were perfused alive with 4 °C normal saline and decollated. Right cerebral tissues were fixed in paraformaldehyde (40 g/L) overnight, embedded and sectioned. Sections were processed for the TUNEL assay using In-Situ Cell-Death Detection (POD) kit, (Roche, Penzberg, Germany) according to the manufacturer’s instructions to reveal the degree of apoptosis in cerebral tissues. The investigators were blinded to the animal grouping. For each rat, four photographs were randomly selected. The apoptotic rate was obtained by counting the TUNEL positive cells compared to all the visible cells. A mean value was calculated from four values and the final values were subjected to statistical analysis.

### Immunohistochemical analysis

Immunohistochemistry (IHC) was carried out with antibodies against BDNF (1:80, Abcam, Cambridge, MA, USA), TrkB (1:100, Abcam, Cambridge, MA, USA), t-PA (H-90) (1:200, Santa Cruz, CA, USA), PAI-1 (H-135) (1:200, Santa Cruz, CA, USA), MMP-2 (8B4) (1:200, Santa Cruz, CA, USA) and MMP-9 (C-20) (1:200, Santa Cruz, CA, USA). The proteins were quantified by integral optical density (IOD) with Image Pro Plus 6.0. For each rat, four sections were used to calculate the means.

### Real-time PCR analysis

Determination of *BDNF* and *TrkB* mRNA was carried out in the hippocampus, *t-PA*, *PAI-1*, *MMP-2* and *MMP-9* mRNA were analyzed in the prefrontal cortex tissue by real-time PCR. Total RNA was extracted using Trizol reagent (Invitrogen, USA) according to the manufacturer’s instructions. Then reverse transcription and real-time PCR reactions were performed with Revert Aid^™^ first strand cDNA synthesis kit (MBI, USA) and SYBR® Green PCR Master Mix (ABI, USA), respectively. The primers were designed with primer 3 web based on published rat cDNA sequences (Table 1). All primers were purchased from SBS Genetech Co., Ltd. (Beijing, China). Quantification of mRNA levels relative to *GAPDH* (a housekeeping gene) was made with the 2^-△△CT^ method.

**Table 1:** Primer pairs used for real-time PCR

Gene	Primer		Product length (bp)
MMP–2	F	5′-ACACCCTCAAGAAGATGCAGA-3′	237
	R	5′-ATACTTTTAAGGCCCGAGCAA-3′	
MMP–9	F	5′-TTTGGAAACGCAAATGGTG-3′	210
	R	5′-TGGAAATACGCAGGGTTTG-3′	
t–PA	F	5′-AGACATCACCTCACACCCTTG-3′	194
	R	5′-TTCCAGGGACCACTCTGTATG-3′	
PAI–1	F	5′-GCCCCACTTCTTCAAGCTC-3′	160
	R	5′-CAGGCGTGTCAGCTCATTT-3′	
BDNF	F	5′-GCATCAGAAAAAGAGGCAAAC-3′	194
	R	5′-AGGCTACGTGAAGTCTTCCAA-3′	
TrkB	F	5′-GTCTGCTGAAGCCTGCATATC-3′	191
	R	5′-ATGAGTGCGTTGGAATGAAAC-3′	
GAPDH	F	5′-CCATGGAGAAGGCTGGG3′	195
	R	5′-CAAAGTTGTCATGGATGACC-3′	

### Western blot analysis

Prefrontal cortex tissues were washed and then lysed on cold RIPA buffer (50 mM Tris–HCl pH 7.4, 150 mM NaCl, 1 % TritonX-100, 1 % sodium deoxycholate, and 0.1 % SDS) containing a protease inhibitor cocktail (Roche, Penzberg, Germany). The protein concentration of each homogenate was determined using a BCA kit. Twenty-four μg of soluble protein was subjected to SDS-PAGE and electro-transferred onto PVDF membranes, which were then immunostained with the following primary antibodies against ZO-1 (1:1000, Thermo Fisher, Rockford, IL, USA), occludin (1:2000, Abcam, Cambridge, MA, USA), claudin-5 (1:1000, Thermo Fisher, Rockford, IL, USA), and β-actin (1:1000, Sigma, St Louis, MO). The membranes were incubated with horseradish peroxidase-conjugated secondary antibodies. Immunoreactive proteins were detected by an enhanced chemiluminescence system.

### Data analysis

The SPSS 16.0 (SPSS Inc., USA) software was used for the analysis. Data were expressed as mean ± standard error. The data were statistically evaluated by one-way analysis of variance, and a post hoc analysis was performed by the Fisher’s least significant difference test. *P* < 0.05 was regarded as statistically significant.

## Results

### Composition of KJ preparation

As shown in Table 2, twelve compounds from the herbs were identified and the content of each compound was in the range of 0.13 to 15.24 mg/g. The contents of hesperidin, naringin, paeoniflorin and nistose were relatively higher among the identified compounds.

**Table 2:** The main bioactive compounds and composition analysis of KJ

Origin of Herb	Compound	Formula	Content (mg/g)
*Morinda officinalis* How	Nistose	C_24_H_42_O_21_	2.50
*Paeonia lactiflora* Pall.	Paeoniflorin	C_23_H_28_O_11_	3.06
*Bupleurum chinense* DC.	Saikosaponin A	C_42_H_68_O_13_	0.28
	Saikosaponin D	C_42_H_68_O_13_	0.13
*Glycyrrhiza uralensis* Fisch.	Glycyrrhizic acid Ammonium salt	C_42_H_65_NO_16_	0.95
	Liquiritin	C_21_H_22_O_9_	0.52
*Citrus aurantium* L.	Naringin	C_27_H_32_O_14_	3.72
	Neohesperidin	C_28_H_34_O_15_	1.06
	Hesperidin	C_28_H_34_O_15_	15.24
*Panax ginseng* C. A. Mey.	Ginsenoside Rg_1_	C_42_H_72_O_14_	1.19
	Ginsenoside Re	C_48_H_82_O_18_	1.01
	Ginsenoside Rb_1_	C_54_H_92_O_23_	1.44

### Behavior test

**Fig. 1:**
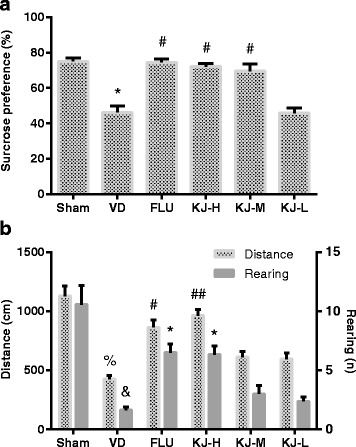
Results of the behavior tests in VD model following KJ treatment. **a** Sucrose preference test. #*P* < 0.05 vs. VD group, *P < 0.05 vs. sham group. **b** Distance and number of rearing in open-field test. #*P* < 0.05, ##*P* < 0.01 vs. VD group, %*P* < 0.01 vs. sham group in distance; *P < 0.05 vs. VD group, &*P* < 0.05 vs. sham group in rearing

SPT and OFT were carried out to investigate the depression-like behavior. VD rats displayed a decreased sucrose preference compared with the sham group (*P* < 0.05); rats in KJ-H, KJ-M and FLU groups had significant increases in sucrose preference compared with the VD group (*P* < 0.05, Fig. 1a). In OFT, VD rats showed a decreased moving distance (*P* < 0.01) and the number of rearing (*P* < 0.05) compared with the sham group; rats in KJ-H and FLU groups had significant increases in the moving distance (*P* < 0.01, *P* <0.05) and number of rearing (*P* < 0.05) compared with the VD group (Fig. 1b).

### Cerebral perfusion

**Fig. 2:**
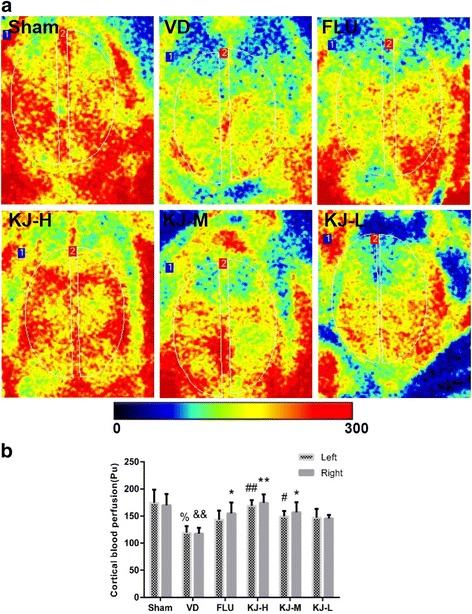
Cortical blood flow in VD model following KJ treatment. **a** the microcirculation imaging of cerebrum. The white line-encircled area is the region of interest. VD rats display a decreased trend of the cortical blood flow (CBF) compared with the sham group. Rats in KJ-H, KJ-M and FLU groups display a notable increase of the CBF. **b** CBF of left and right hemisphere. Pu is a unit of cortical blood flow. #*P* < 0.05, ##*P* < 0.01 vs. VD group; %*P* < 0.01 vs. sham group in the left hemisphere; *P < 0.05, **P < 0.01 vs. VD group; &&*P* < 0.01 vs. sham group in the right hemisphere

The CBF was reduced in VD rats compared with the sham rats (*P* < 0.01); rats in KJ-H and KJ-M groups displayed significant increase in the CBF compared with the VD group (*P* < 0.01, *P <* 0.05, respectively, Fig. 2).

### TUNEL assay

**Fig. 3:**
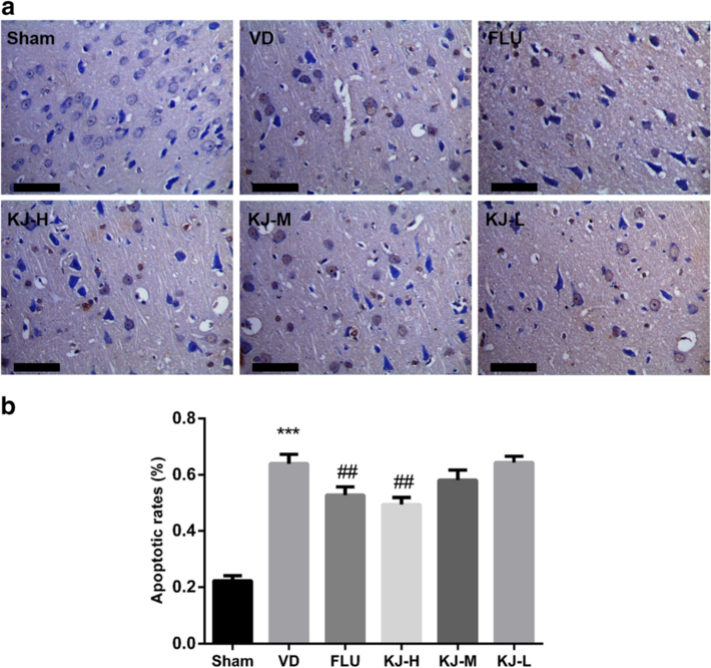
TUNEL assay for analysis of apoptotic cells in brain tissues. **a** TUNEL assay of brain tissues. Cells with shrunken brown stained nuclei were considered positive. The TUNEL-positive cells were mainly observed in cortex. Scale bar: 50 μm. **b** Quantitative analysis of TUNEL-positive cells. ##*P* < 0.01 vs. VD group; ***P < 0.001 vs. sham group

The TUNEL-positive cells were mainly observed in cortex but fewer in hippocampus. More cells were apoptotic in VD rats compared with the sham rats (*P* < 0.001); rats in KJ-H and FLU groups displayed significant decrease in apoptotic rates compared with the VD group (*P* < 0.01, Fig. 3).

### Protein and mRNA of BDNF and TrkB

**Fig. 4:**
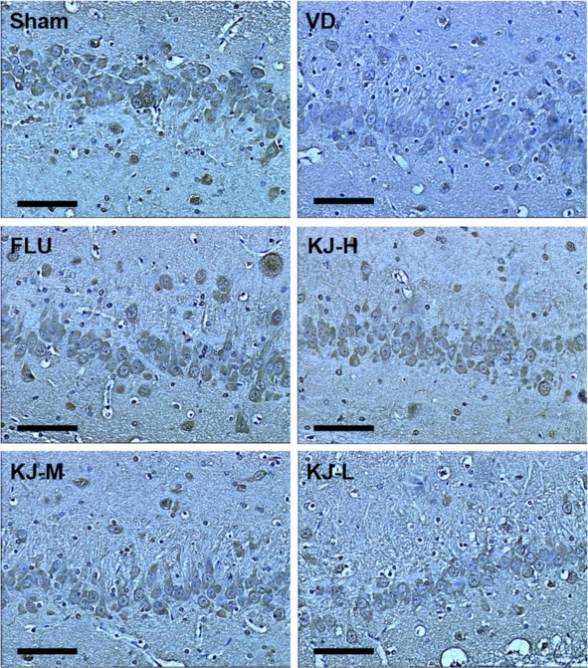
IHC of BDNF protein in the hippocampus. BDNF is mainly expressed in the cytoplasm of neurons and glial cells as yellow or yellow-brown colored staining. More positive-staining cells are in the sham, FLU and KJ-H groups relatively. Scale bar: 50 μm

**Fig. 5:**
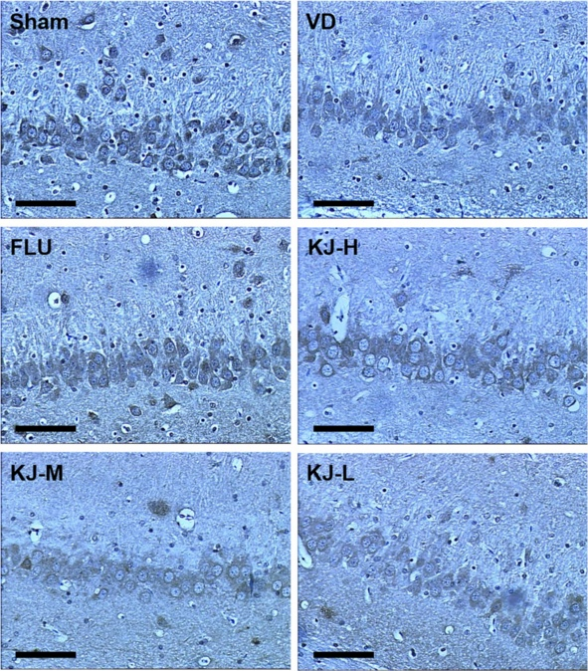
IHC of TrkB protein in the hippocampus. TrkB is mainly expressed in the cytoplasm of neurons and glial cells as yellow or yellow-brown colored staining. More positive-staining cells are in the sham, FLU and KJ-H groups relatively. Scale bar: 50 μm

**Fig. 6:**
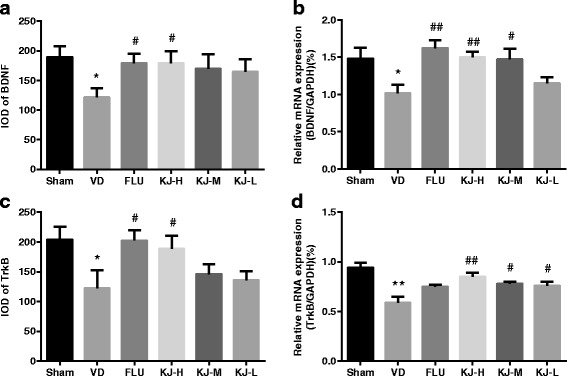
The levels of BDNF and TrkB proteins and mRNA in the hippocampus. **a** IOD of BDNF by IHC. **b** BDNF mRNA levels by real-time PCR. **c** IOD of TrkB by IHC. **d** TrkB mRNA levels by real-time PCR. #*P* < 0.05, ##*P* < 0.01 vs. VD group; **P < 0.01 vs. sham group

IHC-staining showed that BDNF and TrkB were mainly expressed in cytoplasm of neurons and glial cells as yellow or yellow-brown colored staining. BDNF and TrkB in the positive-cells were mainly located in the cytoplasm. The IOD of BDNF and TrkB positive-cells decreased significantly in the VD group compared with the sham group (*P* < 0.05). Compared with VD *g*roup, IOD of BDNF and TrkB positive-cells increased significantly in FLU and KJ-H groups (*P* < 0.05, Figs. 4, 5, 6a and c).

The levels of *BDNF* and *TrkB* mRNA decreased significantly in the VD group (*P* < 0.05, *P* < 0.01, Fig. 6b, d) compared with the sham group. Whereas, compared with VD group, the levels of *BDNF* mRNA increased significantly in KJ-H, KJ-M and FLU groups (*P* < 0.05 or *P* < 0.01, Fig. 6b), *TrkB* mRNA increased significantly in KJ-H, KJ-M and KJ-L groups (*P* < 0.05 or *P* < 0.01, Fig. 6d).

### Protein and mRNA levels of t-PA, PAI-1, MMP-2 and MMP-9

**Fig. 7:**
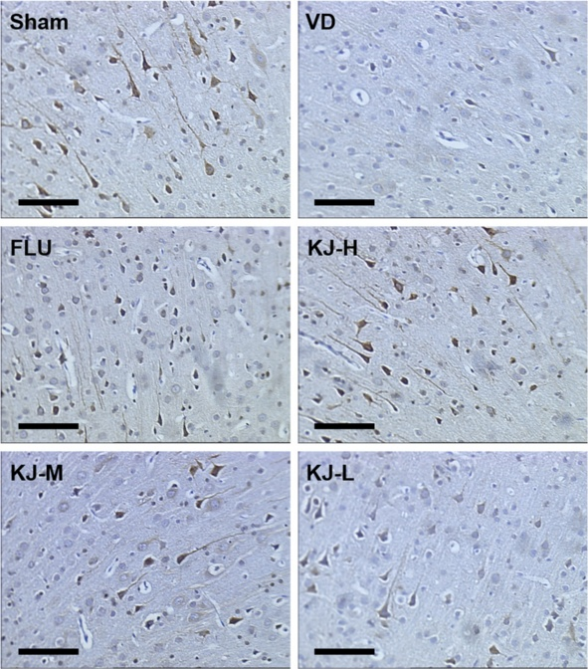
IHC of t-PA protein in the cerebrum. T-PA is expressed mainly in the cytoplasm and a little in the nucleus of neurons and glial cells as yellow or yellow-brown colored staining. More positive-staining cells are in the sham, FLU and KJ-H groups relatively. Scale bar: 50 μm

**Fig. 8:**
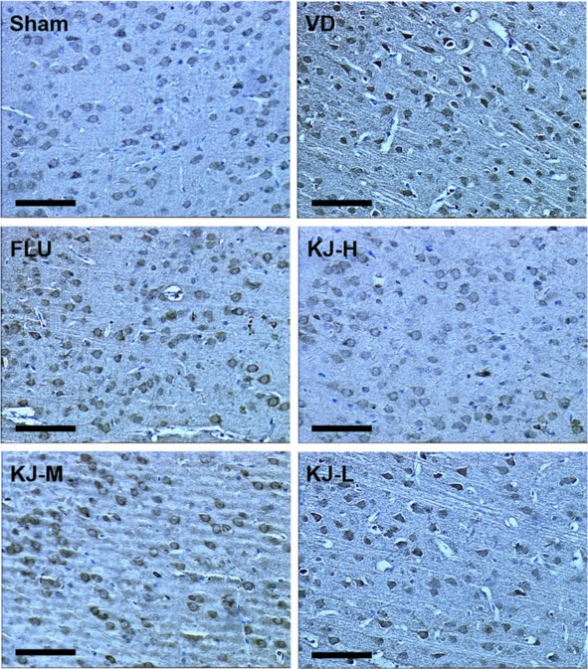
IHC of PAI-1 protein in the cerebrum. PAI-1 is expressed mainly in the cytoplasm and a little in the nucleus of neurons and glial cells as yellow or yellow-brown colored staining. Less positive-staining cells are in the sham and KJ-H groups relatively. Scale bar: 50 μm

**Fig. 9:**
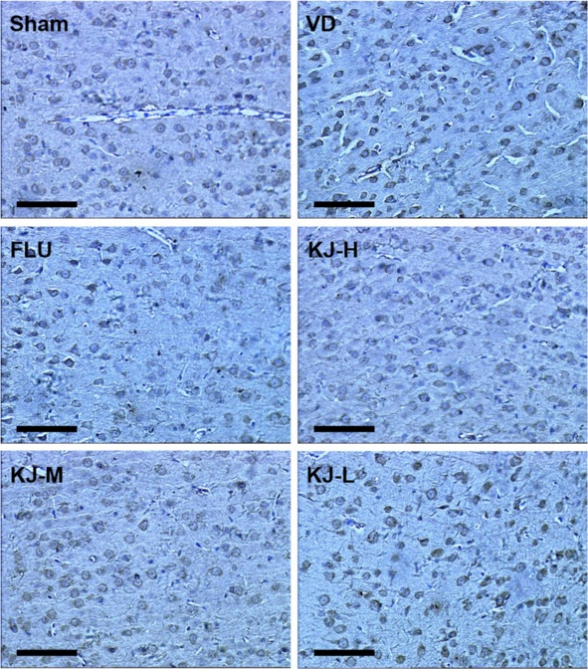
IHC of MMP-2 protein in the cerebrum. MMP-2 is expressed mainly in the cytoplasm and a little in the nucleus of neurons and glial cells as yellow or yellow-brown colored staining. More positive-staining cells are in the VD and KJ-L groups relatively. Scale bar: 50 μm

**Fig. 10:**
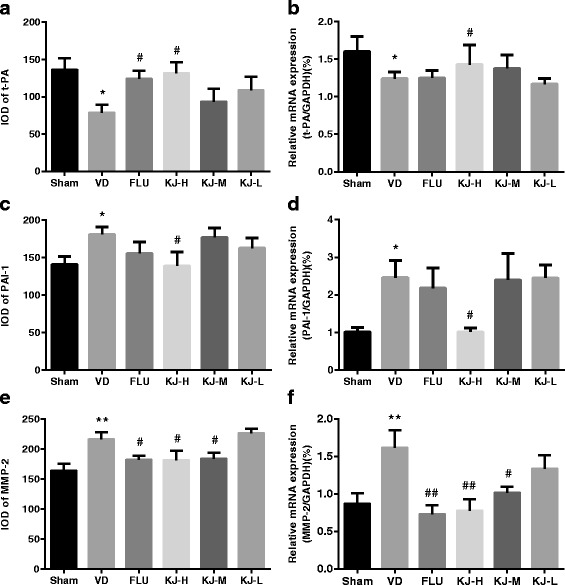
The levels of T-PA, PAI-1and MMP-2 proteins and mRNA in the prefrontal cortex. **a, c, e** IOD of t-PA, PAI-1and MMP-2 by IHC. **b, d, f** T-PA, PAI-1and MMP-2 mRNA levels by real-time PCR. #*P* < 0.05, ##*P* < 0.01 vs. VD group; *P < 0.05, **P < 0.01 vs. sham group

In IHC-stained slices, t-PA, PAI-1, MMP-2 and MMP-9 were expressed mainly in cytoplasm and a little in nucleus of neurons and glial cells as yellow or yellow-brown staining. T-PA and PAI-1 were also slightly expressed in the cytoplasm of endothelial cells. The IOD of t-PA positive-cells decreased significantly, PAI-1 and MMP-2 positive-cells increased significantly in the VD group compared with the sham group (*P* < 0.05 or *P* < 0.01, Figs. 7, 8, 9, 10a, c and e). IOD of t-PA positive-cells increased significantly in KJ-H and FLU groups compared with the VD group (*P* < 0.05, Figs. 7 and 10a); IOD of PAI-1 positive-cells decreased significantly in KJ-H group (*P* < 0.05, Figs. 8 and 10c); IOD of MMP-2 positive cells decreased significantly in KJ-H, KJ-M and FLU groups (*P* < 0.05, Figs. 9 and 10e). There was no significant difference in IOD of MMP-9 positive-cells among all groups.

Compared with the sham group, *t-PA* mRNA decreased significantly (*P* < 0.05), *PAI-1* and *MMP-2* mRNA increased significantly (*P* < 0.05, *P* < 0.01) in the VD group (Fig. 10b, d and f). Levels of *t-PA* mRNA increased and *PAI-1* mRNA decreased significantly in KJ-H group compared with the VD group (*P* < 0.05) (Fig. 10b, d). *MMP-2* mRNA levels decreased significantly in KJ-H, KJ-M and FLU groups (*P* < 0.05 or *P* < 0.01) (Fig. 10f). There was no significant difference in *MMP-9* mRNA levels among all groups.

### Levels of ZO-1, occludin and claudin-5

**Fig. 11:**
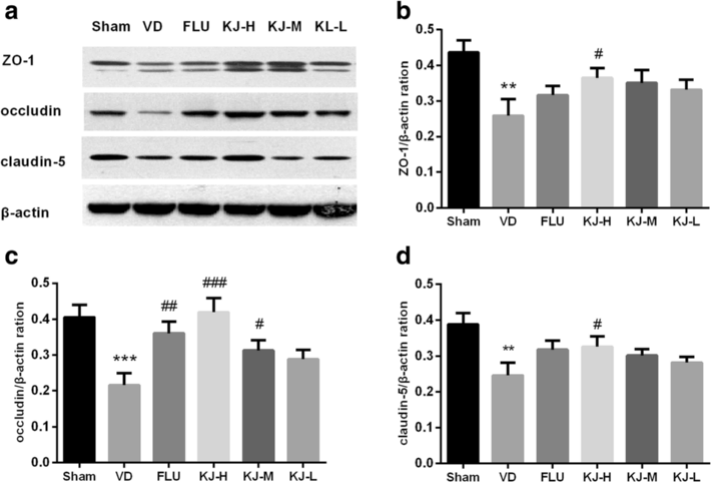
The expression of ZO-1, occludin and claudin-5 proteins in the prefrontal cortex. **a** The expression of ZO-1, occludin and claudin-5 proteins was detected in the prefrontal cortex of VD rats by Western Blot. β-actin was used as a loading control. Relative folds of ZO-1 (**b**), occluding (**c**) and claudin-5 (**d**) were calculated by normalization to β-actin. #*P* < 0.05, ##*P* < 0.01, ###*P* < 0.001 vs. VD group; **P < 0.01, ***P < 0.001 vs. sham group

As shown in Fig. 11a, the expression of tight junction biomarkers including ZO-1, occludin and claudin-5 was significantly decreased in the VD group compared with sham group (*P* < 0.01 or *P* < 0.001). The expression of ZO-1 increased significantly in KJ-H group compared with VD group (*P* < 0.05, Fig. 11b). The expression of occludin increased significantly in KJ-H, KJ-M and FLU groups compared with VD group (*P* < 0.001, *P* < 0.05, *P* < 0.01, Fig. 11c). The expression of claudin-5 increased significantly in KJ-H group compared with VD group (*P* < 0.05, Fig. 11d).

## Discussion

### Effects of KJ on depression-like behavior and cerebral perfusion

A number of rat VD models have been established, such as those resulted from aging [26], high-fat feeding and CUMS [27], individual feeding in a box post LBCCA and treated with CUMS [20]. The VD model established by feeding the rats individually after LBCCA and in combination with CUMS is considered to be ideal in simulating the common pathology (chronic cerebral hypoperfusion caused by cerebrovascular disease) [20]. In the present study, the VD models were evaluated by SPT, OFT and cerebral blood flow. The rats showed increased behavioral obstacles and significantly reduced cerebral perfusion after modelling, indicating that the model was successful in mimicking the core VD symptoms, such as anhedonia, declined athletic activity and reduced curiosity and cerebral perfusion. However, the findings of the present study i.e. the CBF in the VD rats decreased significantly 28 days post LBCCA are different from earlier studies [28, 29], which reported that the perfusion drops sharply immediately after occlusion [28], persists for several days after permanent LBCCA [29]. The compensatory redistribution via the vertebrobasilar network progressively restores the blood flow in almost all brain regions, and the cerebral blood perfusion is indistinguishable from controls after 1 month [28, 29]. The inconsistency suggests that CUMS might increase the duration of cerebral hypoperfusion, and further study is needed to clarify this.

The data demonstrate that that KJ at high and medium doses and FLU have similar antidepressant effects in terms of sucrose preference, moving distance and number of rearing. Furthermore, KJ, particularly at high dose, could improve the cerebral hypoperfusion better than FLU, suggesting it might be a better antidepressant option. The analysis shows that hesperidin (C_28_H_34_O_15_) from *Citrus aurantium* L. is the bioactive compound of the highest-content in KJ preparation. It has been shown to be able to penetrate the BBB [30] and prevent the neurodegeneration caused by conditions such as ischemia/reperfusion and promote healthy brain functions [31]. Therefore, *Citrus aurantium* L. might play a key role in improving the cerebral hypoperfusion and alleviating the depression. Since a number of compounds have been identified in the preparation, more works are needed to elucidate their roles in antidepressant effects.

### Mechanism of KJ on restoration of NVU function

Chronic cerebral hypoperfusion induced by LBCCA is shown to result in hypoxia and ischemia in the brain, leading to impaired BBB [32]. A number of studies have shown that impaired BBB is often associated with depression [33, 34]. Recent studies have shown that oxidative stress and neuro inflammation in depression patients could cause BBB hyperpermeability and NVU dysfunction [17]. To better understand the mechanism of KJ effect on NVU, a number of biomarkers related to neurogenesis, fibrinolytic system and permeability of BBB were investigated at transcriptional and (or) translation levels.

Neurotrophins play important roles in neuron survival, plasticity, neurogenesis and synaptogenesis. BDNF as one of neurotrophins is shown to regulate the neurogenesis in adult hippocampus and generation of 5-hydroxy tryptophan, γ-aminobutyric acid and dopamine in central nervous system, and is involved in the plasticity of neurons [35]. Binding of BDNF to its high-affinity receptor TrkB is found to activate MAPK, PI3-K and CaMK signal pathways, and cAMP response element binding protein and improve the survival and plasticity of neurons [36, 37]. The results show that KJ treatment up-regulates the expression of BDNF and TrkB at translational and transcriptional level and down- regulates the apoptotic rates of neurons in the VD model, suggesting that the antidepressant-like effects of KJ might be attributed to the neurogenesis in the cerebrum.

The t-PA/plasminogen proteolytic cascade is known to be important for thrombolysis [38]. PAI-1 is the major inhibitor for t-PA [39]. Study shows that women with major depressive disorder have higher PAI-1 levels than normal controls [40], and PAI-1 gene variants may play a role in major depressive disorder susceptibility and in the acute therapeutic response to selective serotonin reuptake inhibitors [41]. In addition, PAI-1 could reduce the conversion of plasminogen into plasmin, reduce the cleavage of pro-BDNF to mature BDNF and weaken the protection of nerve by BDNF [42]. This study shows that t-PA in VD rat declines, while PAI-1 increases, resulting in unbalanced fibrinolytic system. KJ could partially restore the fibrinolytic system, which might contribute to improved cerebral perfusion.

The BBB is a tightly sealed barrier between the circulating blood and the central nervous system, consisting of brain microvascular endothelial cells which are characterized by the presence of tight junctions (TJs) and lack of fenestrae, then confer the low paracellular permeability of BBB [43, 44]. The main structural barrier proteins are occludin, zona occludens (ZO)-1 and claudin-5, and these are considered to be sensitive indicators of normal versus disturbed functional states of the BBB [45]. Yet, MMPs have the function of disrupting endothelial junction proteins in BBB *in vitro* and increase BBB opening [46, 47]. Nakaji [48] reported that MMP-2 was associated with BBB permeability and increases the migration of neutrophil and inflammation factors, resulting in damage in the limbic system. Earlier study shows that MMP-9 mediates the damage during ischemia/reperfusion and activation of it occurred at 12, 24 and 48 h after reperfusion [49]. The results show that there is no obvious difference in MMP-9 in the VD rats, which might be because the MMP-9 is mainly involved in the acute phase but not in the chronic phase of cerebral hypoperfusion. The up-regulated levels of MMP-2 protein and mRNA, down-regulated levels of ZO-1, occludin and claudin-5 in the VD rats are partially returned to the normal levels as in the sham group with treatment of KJ. The normalization might reduce the BBB hyperpermeability and inflammation in the VD rats. It might also be responsible for the antidepressant effects of KJ.

## Conclusions

The present study indicated that KJ could reduce depression-like behavior and improve chronic cerebral hypoperfusion in VD rats. The effects might be mediated by the up-regulation of neurogenesis and tight junction of BBB, and balance of the fibrinolytic system, which result in restoration of NVU function. The results imply that KJ is better than FLU in improving cerebral hypoperfusion and the fibrinolytic system.

## Abbreviations

BBB: blood–brain barrier
BDNF: brain-derived neurotrophic factor
CBF: cortical blood flow
CUMS: chronic unpredictable mild stress
FLU: fluoxetine hydrochloride
IHC: immunohistochemistry
IOD: integral optical density
KJ: kaixinjieyu
LBCCA: ligation of bilateral common carotid arteries
MMP: matrix metalloproteinase
NVU: neurovascular unit
OFT: open-field test
PAI-1: plasminogen activator inhibitor-1
SPT: sucrose preference test
t-PA: tissue plasminogen activator
TrkB: tropomyosin receptor kinase B
VD: vascular depression
ZO-1: zona occludens protein-1
